# Successful Treatment of Pure Red Cell Aplasia in Systemic Lupus Erythematosus With a Combination of Rituximab and Mycophenolate Mofetil

**DOI:** 10.7759/cureus.102452

**Published:** 2026-01-28

**Authors:** Maria Carolina Carvalho, João Fernandes Serodio, Marta C Amaral, Susana Oliveira, José Delgado Alves

**Affiliations:** 1 Department of Internal Medicine IV, Hospital Prof. Doutor Fernando Fonseca, Amadora, PRT; 2 Department of Internal Medicine IV, Immune-Mediated Systemic Diseases Unit, Hospital Prof. Doutor Fernando Fonseca, Amadora, PRT

**Keywords:** anaemia, mycophenolate mofetil, pure red cell aplasia, rituximab, systemic lupus erythematosus

## Abstract

Pure red cell aplasia (PRCA) is a rare cause of severe anaemia characterized by selective erythroid suppression in the bone marrow with preservation of other haematopoietic lineages. Its association with systemic lupus erythematosus (SLE) is uncommon and presents significant diagnostic and therapeutic challenges. We describe a 37-year-old female patient with SLE who developed profound isolated anaemia with severe reticulocytopenia, in the absence of other overt clinical manifestations. Laboratory findings revealed active immunological disease, including hypocomplementaemia and elevated anti-double-stranded DNA titres, as well as haemolysis. Although the initial presentation suggested autoimmune haemolytic anaemia, the persistence of reticulocytopenia despite immunosuppressive therapy prompted bone marrow evaluation, which confirmed PRCA. Treatment with high-dose corticosteroids and intravenous immunoglobulin was ineffective, and sustained haematological remission was achieved only after initiation of rituximab, with subsequent incorporation of mycophenolate mofetil (MMF) to maintenance therapy. This case underscores the importance of recognising PRCA as a cause of severe anaemia in SLE and highlights the potential role of B-cell-targeted therapy and MMF as an effective treatment strategy in refractory disease.

## Introduction

Systemic lupus erythematosus (SLE) is a chronic autoimmune disorder classically characterized by variable multisystem involvement and heterogeneous clinical features, presenting with a wide spectrum of severity and requiring individualized treatment approaches. Haematologic abnormalities with peripheral cytopenias are among the most common features of SLE, most often resulting from chronic inflammation, bone marrow suppression, autoimmune destruction, or medication side effects [1]. Anaemia, in particular, may range from mild cases associated with systemic inflammation to severe cases, which are usually due to autoimmune haemolytic anemia (AHAI) [1]. However, in rare instances, severe and potentially life-threatening anaemia in SLE can be caused by pure red cell aplasia (PRCA).

PRCA is a syndrome characterized by normocytic, normochromic anaemia with severe reticulocytopenia, due to a marked reduction or absence of erythroid precursors in the bone marrow, while myeloid and megakaryocytic lineages remain preserved [2]. The condition may be congenital or acquired, and secondary causes of acquired PRCA include thymoma, lymphoproliferative, and autoimmune disorders (e.g., SLE, rheumatoid arthritis, and inflammatory bowel disease), viral infections (particularly parvovirus B19), and medications [2]. Because of its rarity and nonspecific presentation, PRCA in the setting of SLE may be under-recognized, potentially leading to delays in diagnosis and treatment [3].

We present a rare case of PRCA occurring in a patient with SLE, highlighting the diagnostic challenges and therapeutic considerations associated with this unusual manifestation.

## Case presentation

A 37-year-old Caucasian woman with a 12-year history of SLE - initially diagnosed on the basis of arthritis, alopecia, photosensitivity, biopsy-proven subacute cutaneous lupus, positive antinuclear antibodies, anti-SSA, anti-SSB, anti-double-stranded DNA (anti-dsDNA) antibodies, and hypocomplementemia - had been clinically stable on hydroxychloroquine 400 mg/day. She also had a β-thalassemia trait, with a stable baseline haemoglobin level of 10 g/dL.

In March 2024, she developed rapidly worsening anaemia (with haemoglobin values falling to 6.4 g/dL), accompanied by marked reticulocytopenia, while leukocyte and platelet counts remained normal. Initial haemolysis parameters, including lactate dehydrogenase and haptoglobin, were within the normal range (Table 1).

**Table 1:** Initial laboratory findings. ESR, erythrocyte sedimentation rate; dsDNA, double stranded DNA

Laboratory test	Initial results	Reference Range
Haemoglobin	6.4 g/dL	12-15 g/dL
Leukocytes	6700 x 10^9^/L	4000-10,000 x 10^9^/L
Platelets	306 x 10^9^/L	150-400 x 10^9^/L
Reticulocyte count	8000 x 10^9^/L	50,000-100,000 x 10^9^/L
ESR	141 mm	≤10 mm
Haptoglobin	138 mg/dL	30-200 mg/dL
Lactate dehydrogenase	128 U/L	135-214 U/L
Antinuclear antibody	Positive at a titer of 1:640	< 1:40
Anti-dsDNA	Positive (209 UI/mL)	< 10 UI/mL
C3	38 mg/dL	90-180 mg/dL
C4	3.1 mg/dL	10-40 mg/dL
Parvovirus B19 IgG serology	Negative (< 1 UI/mL)	< 2 UI/mL
Parvovirus B19 IgM serology	Negative (<0.10 index)	< 0.9 index

Her haemoglobin levels did not improve despite transfusion with four units of red blood cells. Subsequently, laboratory evidence of haemolysis emerged, and a direct antiglobulin test (DAT) was positive for IgG2 antibodies while remaining negative for IgG1 and IgG3. Notably, reticulocytopenia persisted throughout. Nutritional deficiencies and infectious causes were excluded, including negative parvovirus B19 serology and viral load. The patient denied the introduction of any new pharmacy or recreational drugs or substances.

Further immunological evaluation revealed serological SLE activity, with increased anti-dsDNA titers (209 UI/mL) and markedly reduced complement levels (C3: 38 mg/dL, reference range: 90-180; C4: 3.1 mg/dL, reference range: 10-40). Remarkably, the patient did not show any other symptoms or signs of SLE activity. Bone marrow examination demonstrated profound erythroid hypoplasia, with complete absence of erythroblasts, as well as the presence of lupus erythematosus cells (LE phenomenon), strongly suggesting the diagnosis of PRCA associated with SLE. Complementary imaging studies excluded malignancy, thymoma, and lymphoproliferative disorders.

**Figure 1:**
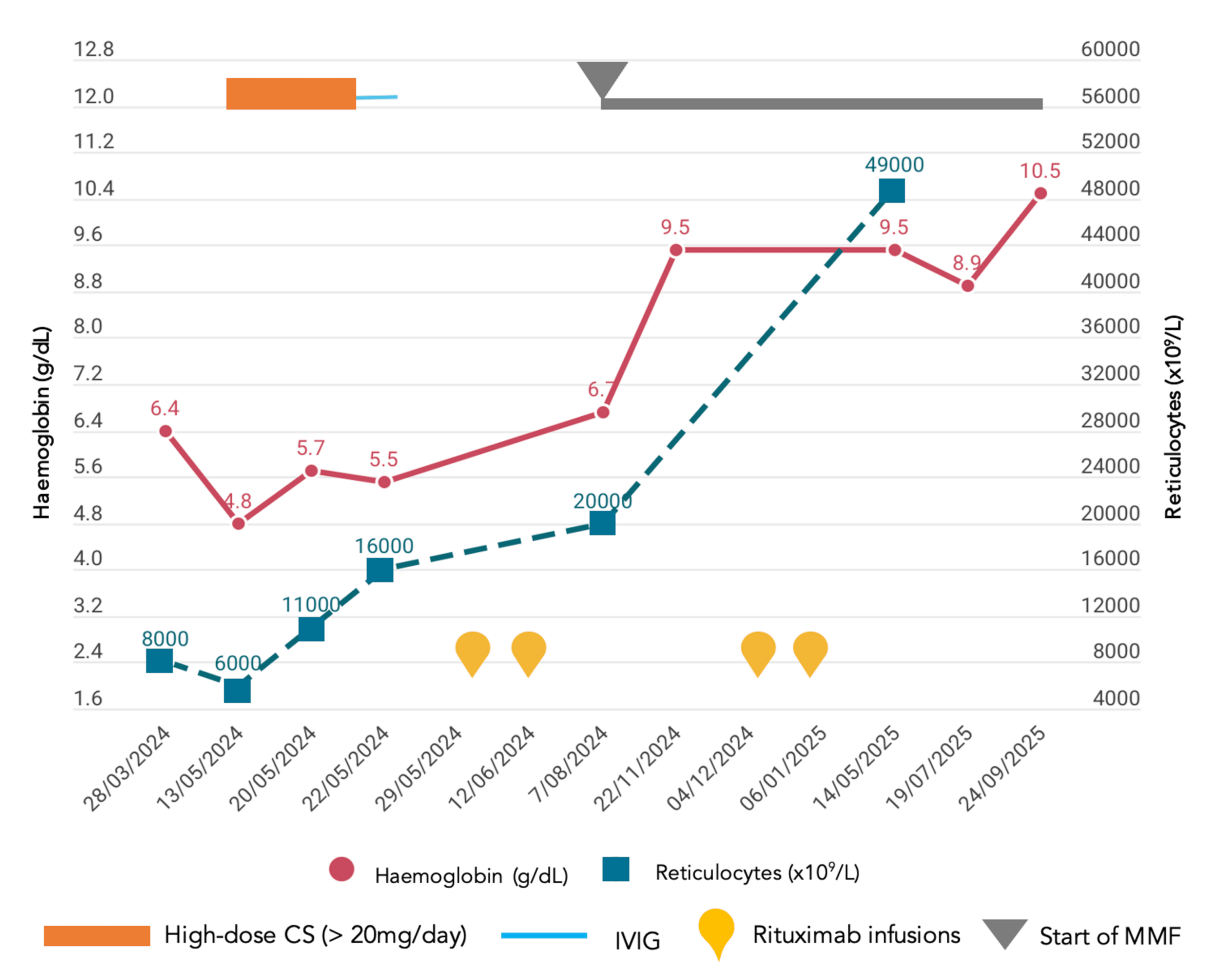
Course of haemoglobin and reticulocyte levels, in relation with implemented therapies. CS, corticosteroid; IVIG, intravenous immunoglobulin; MMF, mycophenolate mofetil

High-dose oral corticosteroid therapy (prednisolone of 1 mg/kg/day) was initiated without benefit, and haemoglobin levels further deteriorated to 4.8 g/dL, accompanied by severe fatigue. Escalation of therapy with methylprednisolone (500 mg pulses for three days), followed by intravenous immunoglobulin (IVIG), 0.4 g/kg for five days, led to normalization of complement and haptoglobin levels but failed to improve anaemia or reticulocyte counts, and the patient remained dependent on transfusional support. Given the refractory course, rituximab was initiated (two 1 g doses administered 14 days apart), resulting in a favourable haematologic response, with progressive recovery of haemoglobin levels to baseline values and allowing corticosteroid tapering (Figure 1).

Two months later, early B-cell repopulation was observed, prompting the addition of mycophenolate mofetil (MMF) at 2 g/day to maintain remission. Rituximab retreatment was administered six months later, with sustained efficacy and no significant adverse events. One year after the last rituximab infusion, the patient remains in complete haematological remission on MMF of 1 g/day, prednisolone of 5 mg/day, and hydroxychloroquine, with normalization of complement levels and decreased anti-dsDNA titres.

## Discussion

Haematological manifestations in SLE are extremely common [4]. Although lymphopenia is the most frequently observed cytopenia, anaemia affects over 50% of patients during the course of the disease and is typically multifactorial, arising from both immune- and non-immune-mediated mechanisms [5]. The most frequent causes include anaemia of chronic disease, iron deficiency, autoimmune haemolysis, and iatrogenic myelotoxicity related to drugs such as azathioprine. However, bone marrow involvement may also occur, manifesting as aplastic anaemia, myelofibrosis, or, as in the present case, PRCA [5,6].

As previously stated, PRCA is characterized by selective suppression of erythropoiesis in the bone marrow, resulting in profound reticulocytopenia (<10 x 10^9^/L), while other haematopoietic lineages remain preserved [2]. Its association with SLE is rare, and reported prevalence estimates are low, with most available evidence deriving from isolated case reports and small case series [3].

While the pathogenesis of SLE-associated PRCA is not fully understood, proposed mechanisms include antibody or T-cell-mediated inhibition or destruction of erythroid precursors, with some case reports describing the presence of anti-erythropoietin autoantibodies [7]. In this case, the presence of lupus erythematosus cells in the bone marrow supports an immune-mediated mechanism driven by SLE activity. This is further reinforced by the concomitant rise in anti-dsDNA titres and hypocomplementaemia, despite the absence of other overt systemic manifestations of disease activity. Similarly, in a case series of 24 patients with PRCA and SLE, those with a prior diagnosis of SLE often had clinically inactive disease at the time of PRCA onset, with no clear correlation with other symptoms [8].

When confronted with isolated and profound anaemia in a patient with SLE, particularly in the absence of other clinical features, the principal differential diagnosis is AIHA, which occurs in 5-10% of cases [6]. Distinguishing PRCA from AIHA may be challenging, particularly because PRCA can occasionally present with laboratory evidence of haemolysis and a positive DAT, as observed in the present case, thereby adding to the diagnostic complexity [3,7]. The key feature that should prompt consideration of PRCA is the coexistence of severe and persistent reticulocytopenia, in contrast to the reticulocytosis typically seen in AIHA [1]. In our patient, the persistence of reticulocytopenia despite immunosuppression was a crucial diagnostic clue, ultimately leading to bone marrow examination and confirmation of profound erythroid hypoplasia.

Another useful discriminator between PRCA and AIHA is the therapeutic response to corticosteroids. AIHA is usually characterized by a favourable response to corticosteroid therapy, with only approximately 10% of patients requiring a second-line treatment [1]. In contrast, the limited number of case reports and small case series describing PRCA in SLE suggest a poor response to corticosteroids, with up to 79% of patients requiring additional immunosuppressive therapy [9].

Since PRCA is itself a rare haematological condition with multiple causes, the underlying pathophysiological mechanisms vary and therefore require distinct therapeutic approaches [10,11]. Responsiveness to IVIG is most commonly associated with parvovirus B19 infection; however, favourable responses have also been reported in cases of PRCA associated with hypogammaglobulinaemia, with or without concomitant thymoma [10]. In the setting of SLE, therapeutic strategies are largely guided by anecdotal evidence and extrapolated from idiopathic PRCA. Intensive immunosuppression with agents such as cyclophosphamide and cyclosporine is frequently employed, but the risks associated with these therapies underscore the need for other treatment strategies [12-18]. Notably, several reports have described successful treatment of SLE-associated PRCA with plasmapheresis, suggesting that autoantibodies against erythropoietin or erythroid progenitors may indeed play a central role in the pathogenesis of this association [14]. In our patient, high-dose corticosteroids and IVIG appeared to curb lupus activity, as reflected by the normalization of complement levels, but failed to correct the anaemia, highlighting the refractory nature of this condition. Remission was achieved only after the introduction of rituximab; incomplete B-cell depletion subsequently prompted the addition of MMF. Rituximab has emerged as a valuable therapeutic option in immune-mediated cytopenias associated with SLE, including AIHA and immune thrombocytopenia; however, its role in PRCA is less well defined but increasingly reported [19]. MMF has also been described as efficacious in autoimmune cytopenias in SLE, although its role in PRCA is less clear [20]. In this case, rituximab induced a sustained haematological remission, allowing corticosteroid tapering and long-term disease stabilization along with MMF. This response supports a pathogenic role for B-cell-mediated autoimmunity in SLE-associated PRCA and suggests that B-cell-targeted therapy may represent an effective strategy in refractory disease.

## Conclusions

PRCA is a rare and diagnostically challenging cause of severe anaemia in SLE and may occur even in the absence of other overt manifestations of disease activity. This case highlights the importance of recognizing persistent reticulocytopenia as a key diagnostic clue that should prompt consideration of PRCA and timely bone marrow evaluation, thereby reducing diagnostic delays.

Accurate recognition of SLE-associated PRCA is essential, as it is frequently refractory to conventional therapies, such as corticosteroids and intravenous immunoglobulin, in contrast to autoimmune haemolytic anaemia. The optimal management of this clinical entity remains uncertain, and reported treatment strategies are heterogeneous and based on heavy immunosuppression. In this case, the innovative and successful use of rituximab combined with MMF suggests a promising and well-tolerated therapeutic strategy for refractory PRCA associated with SLE, warranting further investigation. Until more robust evidence becomes available, careful clinical assessment and an individualised therapeutic strategy remain central to achieving favourable outcomes.
